# Predicting Preterm Birth with Strain Ratio Analysis of the Internal Cervical Os: A Prospective Study

**DOI:** 10.3390/jcm12123885

**Published:** 2023-06-07

**Authors:** Alina-Madalina Luca, Raluca Haba, Luiza-Maria Cobzeanu, Dragos Nemescu, Anamaria Harabor, Raluca Mogos, Ana-Maria Adam, Valeriu Harabor, Aurel Nechita, Gigi Adam, Alexandru Carauleanu, Sadiye-Ioana Scripcariu, Ingrid-Andrada Vasilache, Tudor Gisca, Demetra Socolov

**Affiliations:** 1 Department of Obstetrics and Gynecology, ‘Grigore T. Popa’ University of Medicine and Pharmacy, 700115 Iasi, Romania; 2 Surgical Department, Faculty of Medicine, University of Medicine and Pharmacy “Grigore T. Popa”, 700115 Iasi, Romania; 3 Clinical and Surgical Department, Faculty of Medicine and Pharmacy, ‘Dunarea de Jos’ University, 800216 Galati, Romania; 4 Department of Pharmaceutical Sciences, Faculty of Medicine and Pharmacy, ‘Dunarea de Jos’ University, 800216 Galati, Romania; gigi.adam@ugal.ro

**Keywords:** cervix, elastography, preterm birth, prediction, performance

## Abstract

(1) Background: Cervical elastography is a new concept that could allow clinicians to assess cervical consistency in various clinical scenarios. We aimed to evaluate the predictive performance of the strain ratio (SR) at the level of the internal os, either individually or in combination with other parameters, in the prediction of spontaneous preterm birth (PTB) at various gestational ages. (2) Methods: This prospective study included 114 pregnant patients with a high-risk profile for PTB who underwent cervical elastography during the second trimester. Clinical and paraclinical data were assessed using univariate analysis, logistic regression, and sensitivity analysis. (3) Results: The SR achieved an area under the receiver operating curve (AUROC) value of 0.850, a sensitivity of 85.71%, and a specificity of 84.31% in the prediction of PTB before 37 weeks of gestation. The combined model showed superior results in terms of accuracy (AUROC = 0.938), sensitivity (92.31%), and specificity (95.16%). When considering PTB subtypes, the highest AUROC value (0.80) and accuracy (95.61%) of this marker were achieved in the prediction of extremely preterm birth, before 28 weeks of gestation. (4) Conclusions: The SR achieved an overall good predictive performance in the prediction of PTB and could be further evaluated in various cohorts of patients.

## 1. Introduction

The uterine cervix plays a crucial role in supporting pregnancy until delivery. The cervix’s ability to adapt to the expanding volume of the uterine contents and increasing pressure is contingent on its length and firmness, and the closure of its internal orifice. The mechanical characteristics of the cervix undergo modifications during the course of pregnancy. Preceding parturition, the cervix undergoes a process of ripening, followed by effacement and dilation in response to uterine contractions. Inadequate cervical ripening can lead to premature delivery, with important consequences for neonatal outcomes.

Recently, cervical elastography has emerged as a new concept that can allow clinicians to assess cervical consistency. The theoretical basis of elastography is founded on the measurement of the displacement between two points within an organ when external pressure is applied [1]. The tissue can be characterized as hard and rigid when there is either no alteration in distance or a negligible change. Soft and dilatable tissue exhibits notable alterations [2]. The determination of tissue consistency is denoted as strain, whereby an increase in strain is indicative of lower tissue firmness, and conversely, a decrease in strain is associated with higher tissue firmness.

Several studies have outlined the applicability of cervical elastography for the prediction of preterm birth (PTB) [3,4,5]. A prospective study by Woźniak et al., on 109 pregnant patients with a short cervix evaluated during the second trimester scan, showed that preterm birth was associated with softening of the internal cervical orifice [6]. Moreover, Hernandez-Andrade et al. reported comparable findings in a cross-sectional study of 545 patients with a low risk of preterm birth that evaluated the values of the strain ratio for quartiles of the endocervical region and whole cervix [7]. Specifically, the authors reported a positive correlation between an increased strain ratio in the internal os region and the risk of preterm delivery, but without significant correlations between elastography parameters of the complete cervix or external os and preterm birth.

Several algorithms for the prediction of preterm birth, based on maternal risk factors, ultrasound markers, and serum biomarkers, have been evaluated in the current literature [8,9]. Among the most commonly associated maternal risk factors for PTB are the following: age, ethnicity, obesity, smoking during pregnancy, assisted reproductive techniques, genital and urinary tract infections, autoimmune disorders, thrombotic disorders, gestational diabetes and preeclampsia, and personal history of adverse pregnancy outcomes (recurrent pregnancy loss, history of PTB, etc.) [10,11,12,13,14]. The predictive performance of algorithms that are solely based on maternal risk factors is modest, with an area under the curve (AUC) value of 0.67, as reported by Damaso et al. [10].

Ultrasound parameters such as a short cervical length and the pulsatility index of the uterine artery have also been assessed from the perspective of PTB, but the results have been conflicting [15,16,17]. In a secondary analysis conducted by Grobman and colleagues, a short cervical length (<30 mm) on transvaginal ultrasound in the second trimester, between 16 and 22 weeks of gestation, achieved an AUC value of only 0.63 in the prediction of spontaneous PTB before 34 weeks of gestation [18].

Recently, some authors have questioned the predictive performance of a short cervical length of less than 30 mm compared to that of less than 25 mm and suggested that a cut-off of 30 mm in the second trimester of pregnancy may more accurately predict the risk of PTB before 35 and 37 weeks of gestation (AUC: 0.70) [19]. However, the majority of observational studies reported a cut-off of 2.5 cm [20,21,22], which demonstrated a moderate predictive performance.

Finally, numerous biomarkers have been studied for the prediction of PTB, but only a few, such as cervical fetal fibronectin, alpha fetoprotein, C-reactive protein, and interleukin 6, have obtained good results in terms of the predictive performance in spontaneous preterm birth [23,24].

A recent study by Jung et al. demonstrated that the addition of cervical elastography parameters to the cervical length determined via transvaginal ultrasound can improve the overall prediction of spontaneous preterm birth before 32 weeks of gestation [3]. The aim of this study was to evaluate the predictive performance of the strain ratio measured at the level of the internal os in the prediction of spontaneous preterm birth. A secondary aim of this study was to evaluate the predictive performance of this parameter in combination with the cervical length and clinical risk factors for PTB and its subtypes.

## 2. Materials and Methods

This prospective study included 114 pregnant patients who attended the Clinical Hospital of Obstetrics and Gynecology “Cuza-Voda”, Iasi, Romania, between July 2021 and January 2023, with a high-risk profile for PTB either due to a short cervical length (less than 2.5 cm) or due to the presence of at least two risk factors for PTB. The patients were enrolled in this study during the second trimester anatomy scan, between 18 and 24 weeks of gestation. Informed consent was obtained from all participants, and ethical approval for this study was obtained from the Institutional Ethics Committee of the University of Medicine and Pharmacy “Grigore T. Popa” (No. 101/8 July 2021).

The inclusion criteria were as follows: patients aged between 18 and 45 years with singleton pregnancies, between 18 and 24 weeks of gestation at enrollment, who had a history of adverse pregnancy outcomes (history of preterm birth, recurrent pregnancy loss, stillbirth, ischemic placental disease, or cervical insufficiency) or a cervical length less than 2.5 cm as determined via transvaginal ultrasound, and who offered their informed consent to participate in this study. The exclusion criteria comprised underage patients, twin pregnancies, fetal chromosomal and structural abnormalities, history of cervical surgery such as cervical conization or loop electrosurgical excision procedures, diagnosis of placenta accreta spectrum disorders, incomplete medical data, or inability to provide informed consent.

The patients were subjected to a detailed assessment of their medical history and current medical conditions, along with a thorough clinical examination. The study documented several variables, including age, medium, body mass index (BMI), smoking status, gestational age at enrollment, parity, adverse pregnancy outcomes (such as preterm birth, preeclampsia, intrauterine growth restriction, and stillbirth), and comorbidities.

The patients underwent standard anatomy scans as well as transvaginal ultrasounds performed by experienced obstetricians, with at least a level 2 qualification in ultrasound examination, using an E10 scanner with a 4.8 MHz transabdominal probe, and a 5–15 MHz intravaginal probe (GE Medical Systems, Milwaukee, WI, USA).

**Figure 1:**
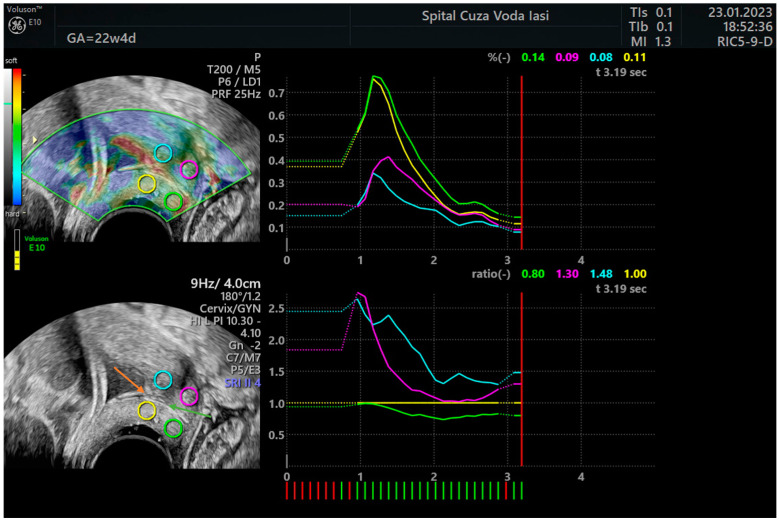
Cervical elastography with the dynamic measurement of the strain ratio in the first 3 cycles of compression–decompression (first example). Legend: orange arrow—internal cervical os; green arrow—cervical canal; blue ring—region of interest on the anterior lip of the internal cervical os; yellow ring—region of interest on the posterior lip of the internal cervical os; pink ring—reference region on the anterior lip of the external cervical os; green ring—reference region on the posterior lip of the external cervical os.

**Figure 2:**
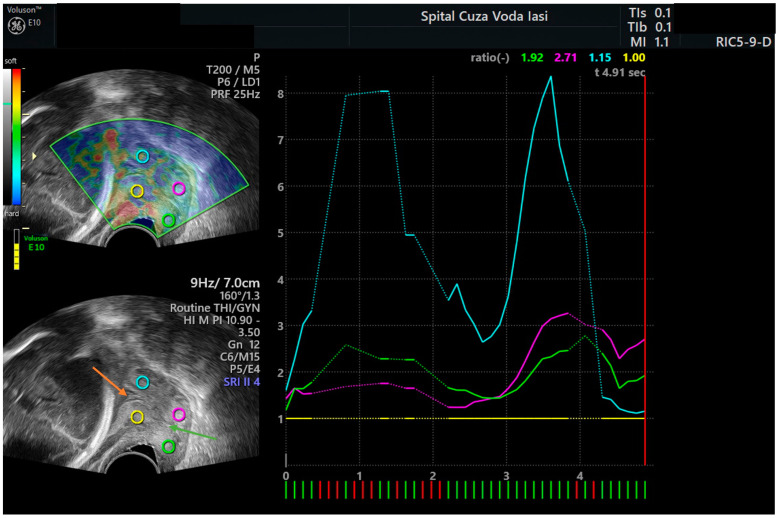
Cervical elastography with the measurement of the strain ratio after 5 cycles of compression–decompression (second example). The mean strain ratio of the internal cervical os was considered for further analysis. Legend: orange arrow—internal cervical os; green arrow—cervical canal; blue ring—region of interest on the anterior lip of the internal cervical os; yellow ring—region of interest on the posterior lip of the internal cervical os; pink ring—reference region on the anterior lip of the external cervical os; green ring—reference region on the posterior lip of the external cervical os.

The participants were instructed to empty their bladders and to adopt the lithotomy position for the cervical elastography examination. A transvaginal probe was inserted into the anterior vaginal fornix to locate the bladder as a reference point. Subsequently, a conventional sagittal image of the cervix was acquired, and the cervical length was determined as the distance between the internal cervical os and external cervical os. The probe was utilized to generate a maximum of five compression and decompression cycles while operating in elastography mode. We obtained a sagittal section of the cervix and marked the regions of interest (ROIs) at the level of the internal cervical os as reported by Hernandez-Andrade et al. [7]: region of interest on the anterior lip of the internal cervical os, region of interest on the posterior lip of the internal cervical os, reference region on the anterior lip of the external cervical os, and reference region on the posterior lip of the external cervical os (see examples in Figure 1 and Figure 2). The Elastography Analysis program was used to calculate numerical values for the strain ratio based on the selected regions during 5 cycles of compression–decompression, and the mean recorded values of the strain ratios in the regions of interest corresponded to mean recorded value of the internal cervical os strain ratio.

The patients were followed up until delivery, and pregnancy outcomes were recorded. Depending on the gestational age at delivery, the patients were segregated into the following groups: group 1 (*n* = 63 patients) comprised patients who delivered before term (before 37 completed weeks of gestation), and group 2 (*n* = 51 patients) comprised patients who delivered at term and were considered controls.

The evaluated pregnancy outcomes were gestational age at birth, type of birth, newborn’s gender, Apgar score, neonatal intensive care unit (NICU) admission, fetal death, and the need for mechanical ventilation.

In the first stage of the statistical analysis, categorical variables were evaluated with chi-squared and Fisher exact tests, which are presented as frequencies with the corresponding percentages, and continuous variables were evaluated with *t*-tests, which are presented as means and standard deviations (SD).

In the second stage of the analysis, we evaluated and compared the predictive performance of the strain ratio and a combined algorithm using logistic regression and ROC analysis. Cut-off values, adjusted to gestational age at diagnosis, were identified according to the Youden index. The combined algorithm comprised the values of the strain ratio, cervical length, and maternal characteristics recorded during the prenatal visit in the second trimester (maternal age, BMI, smoking status, previous history of preterm birth, gestational diabetes, pregnancy-induced hypertension, vaginal or urinary tract infections).

The classification systems for PTB are heterogeneously reported in the literature [25]. The WHO classifies this concept as follows: extremely PTB (<28 weeks of gestation), very PTB (between 28 and 31 + 6 weeks of gestation), moderate PTB (between 32 and 33 + 6 weeks of gestation), and late PTB (between 34 and 36 + 6 weeks of gestation) [26]. Since our cohort of patients was small, we decided to evaluate the predictive performance of the SR and combined model considering the following subtypes of preterm birth: late preterm birth (between 34 and 37 weeks of gestation), early preterm birth (between 28 and 34 weeks of gestation), and extremely preterm birth (before 28 completed weeks of gestation).

The statistical analyses were performed using STATA SE (version 17, 2022, StataCorp LLC, College Station, TX, USA). A *p* value less than 0.05 was considered statistically significant.

## 3. Results

A total of 114 pregnant patients were included in this prospective study, segregated into the following groups: group 1 (*n* = 63 patients, preterm birth) and group 2 (*n* = 51 patients, controls). Their clinical and demographic characteristics are presented in Table 1. The pregnant patients who delivered prematurely presented a significant personal history of preterm birth (*p* < 0.001) and other adverse pregnancy outcomes (*p* < 0.001), as well as a shorter cervical length (*p* = 0.03), in comparison with controls.

Cervical funneling was discovered in eight patients who delivered before term (12.69%, *p* = 0.03). On the other hand, the control group had a significantly higher primiparity rate in comparison with the first group (*p* < 0.001).

Regarding pregnancy and neonatal outcomes, we determined significantly higher rates of NICU admission (*p* = 0.02) and invasive ventilation (*p* = 0.03) in the preterm group compared to controls (Table 2). Moreover, the birthweight (*p* < 0.001) and Apgar score at 1 min (*p* = 0.003) were significantly lower compared with controls. Two neonatal deaths were recorded in this cohort of patients due to extreme prematurity.

**Figure 3:**
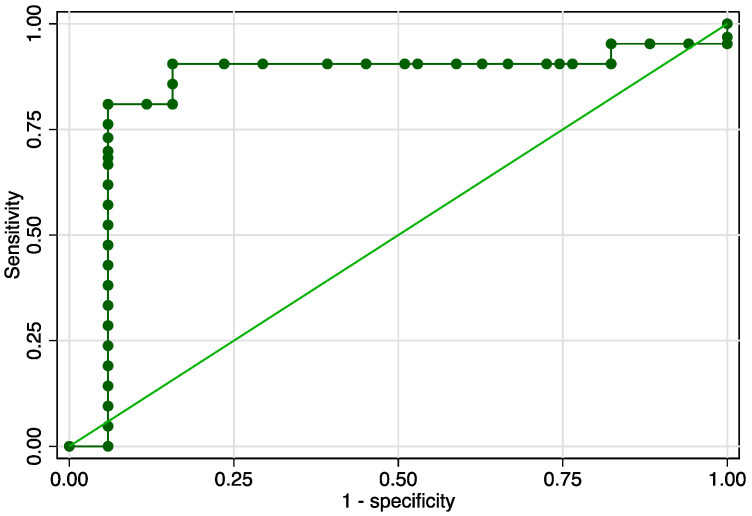
Graphical representation of area under the ROC for the SR at the internal cervical os.

**Figure 4:**
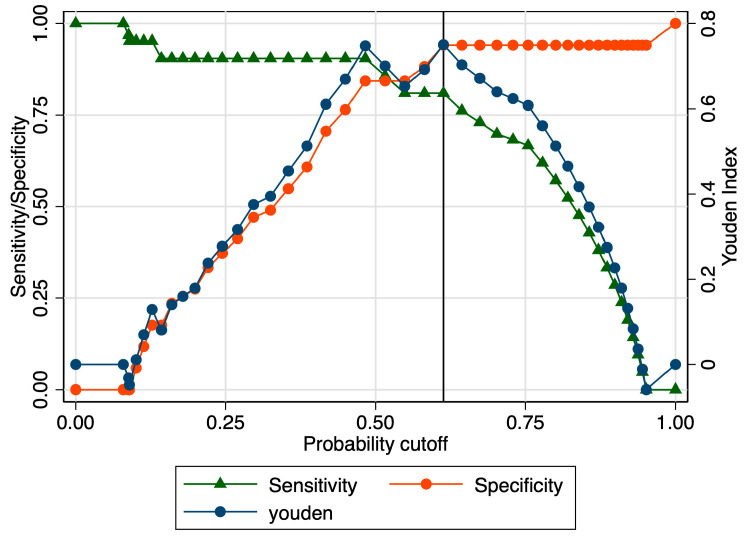
Graphical representation of the Youden Index for the SR at the internal cervical os.

**Figure 5:**
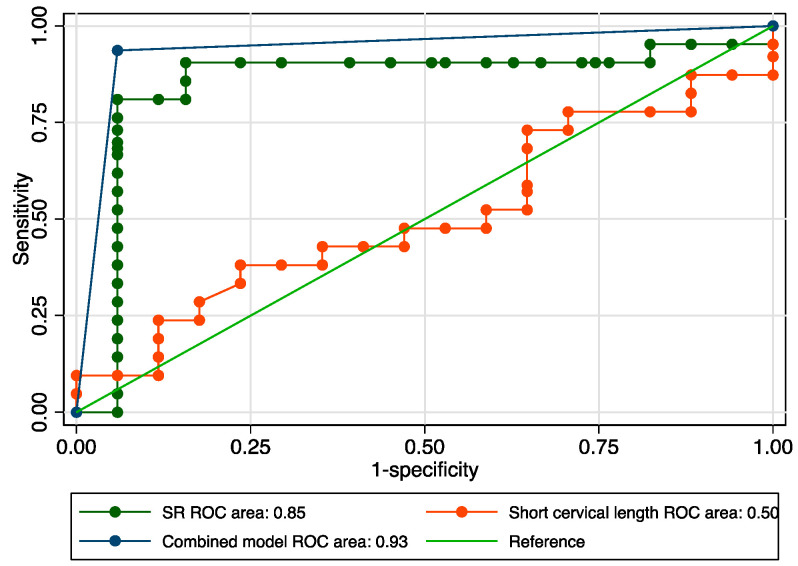
Graphical representation of the comparison between ROC curves for the SR, short cervical length, and combined model.

The sensitivity analysis revealed an area under the receiver operating curve (ROC) value of 0.850 (Figure 3), considering a determined cut-off of 0.93 for the SR (Figure 4). The corresponding values for sensitivity, specificity, and precision were 85.71%, 84.31%, and 87.10%, respectively (Table 3). Cervical length achieved a modest value for the AUROC: 0.55 (Figure 5). The combined model, which comprised SR values, cervical length, and the presence of maternal risk factors, showed superior results in terms of accuracy (area under ROC = 0.9388), sensitivity (92.31%), specificity (95.16%), and precision (94.12%) (Figure 5 and Table 3).

**Figure 6:**
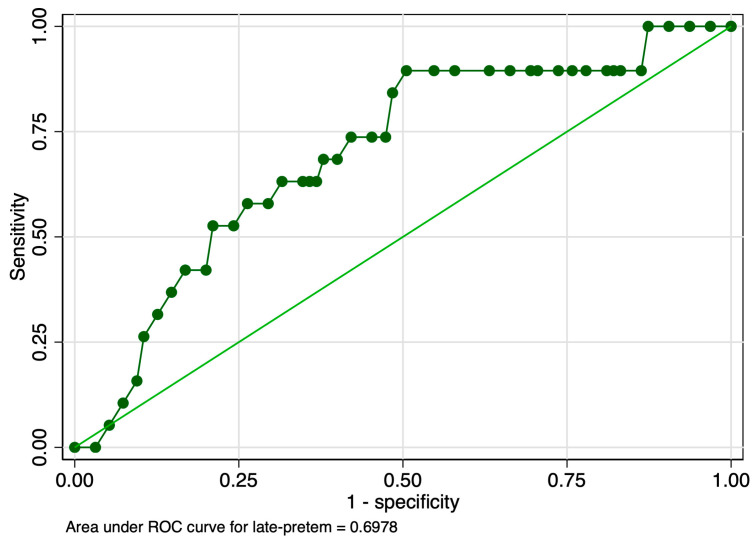
Graphical representation of the area under the ROC for the SR at the internal cervical os considering the late preterm category.

**Figure 7:**
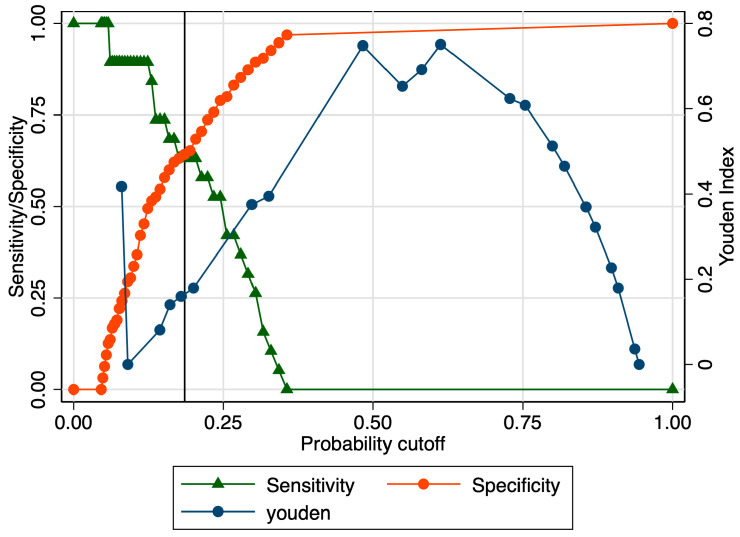
Graphical representation of the Youden Index for the SR at the internal cervical os considering the late preterm category.

**Figure 8:**
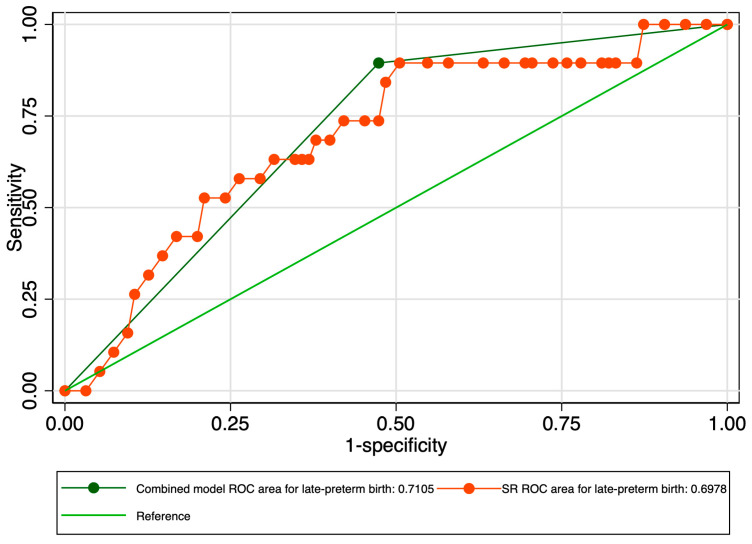
Graphical representation of the comparison between ROC curves for the SR and combined model considering the late preterm category.

When evaluating the predictive performance of the strain ratio considering the subtypes of preterm birth, our results revealed an AUROC value of 0.69 in the prediction of late preterm birth (Figure 6). The calculated probability cut-off using the Youden index was 0.58 (Figure 7), and the determined cut-off for this elastography marker using sensitivity analysis was 2.56 (Table 3). The corresponding values for sensitivity, specificity, and precision were 5.2%, 98.9%, and 50%, indicating a poor overall predictive performance (Table 3). The combined model showed slightly better results in terms of the AUROC (0.71), sensitivity (96.15%), specificity (24.42%), and precision (52.63%), but the overall accuracy was low (58.1%) (Figure 8 and Table 3).

**Figure 9:**
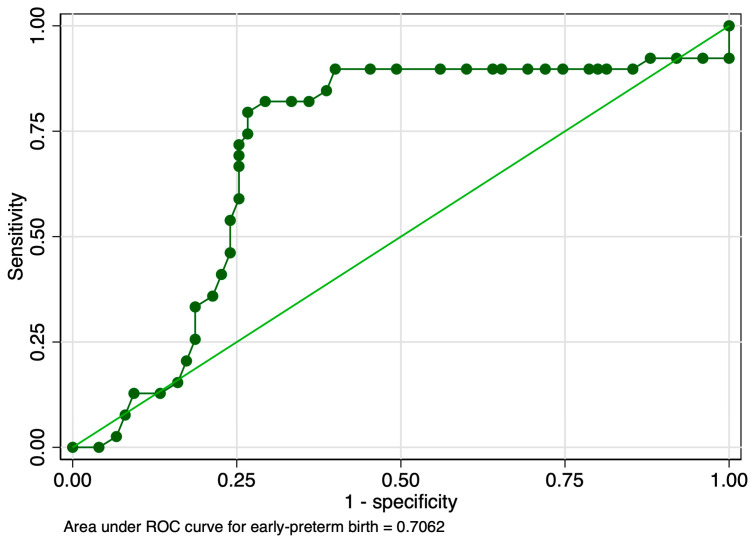
Graphical representation of the area under the ROC for the SR at the internal cervical os considering the early preterm category.

**Figure 10:**
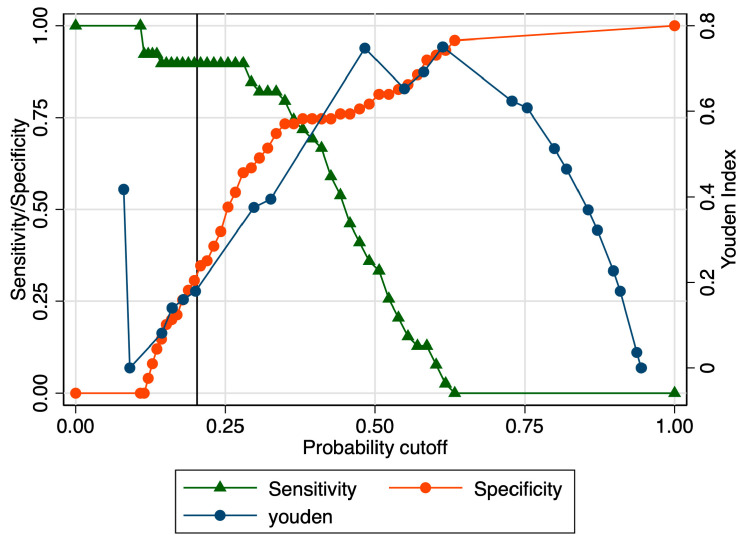
Graphical representation of the Youden Index for the SR at the internal cervical os considering the early preterm category.

**Figure 11:**
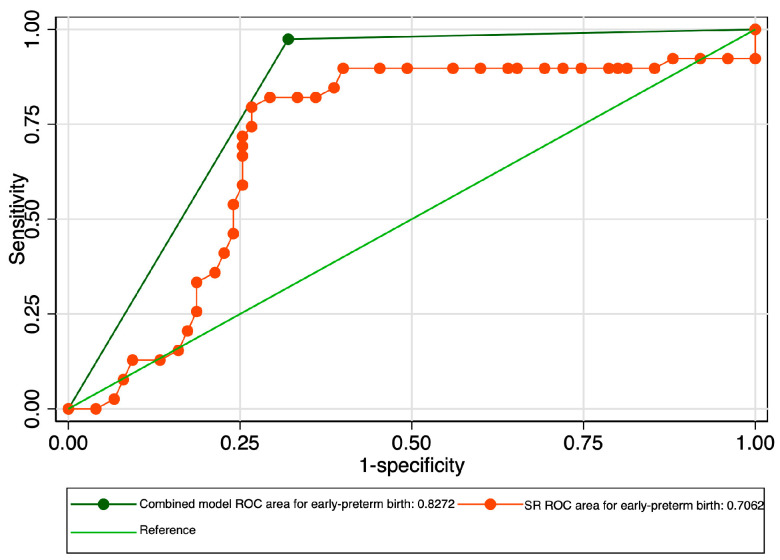
Graphical representation of the comparison between ROC curves for the SR and combined model considering the early preterm category.

When evaluating the predictive performance of the strain ratio at the level of the internal cervical os in the prediction of early preterm birth, it achieved an AUROC value of 0.70 (Figure 9). The calculated probability cut-off using the Youden index was 0.53 (Figure 10), and the determined cut-off for this elastography marker using sensitivity analysis was 1.66 (Table 3). The corresponding values for sensitivity, specificity, and precision were 33.33%, 81.33%, and 48.15%, respectively (Table 3). The combined model showed an improved performance in terms of the AUROC (0.82), sensitivity (98.08%), specificity (61.29%), and precision (68%), with a good overall accuracy (78.07%) (Figure 11 and Table 3).

**Figure 12:**
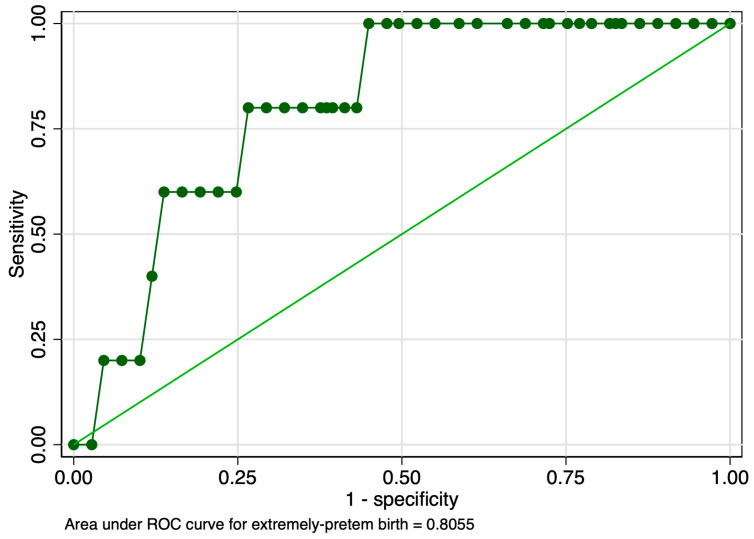
Graphical representation of the area under the ROC for the SR at the internal cervical os considering the extremely preterm category.

**Figure 13:**
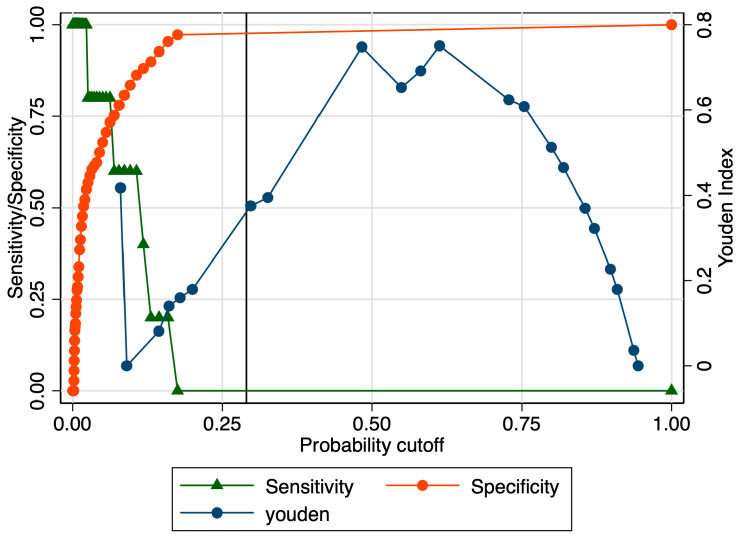
Graphical representation of the Youden Index for the SR at the internal cervical os considering the extremely preterm category.

**Figure 14:**
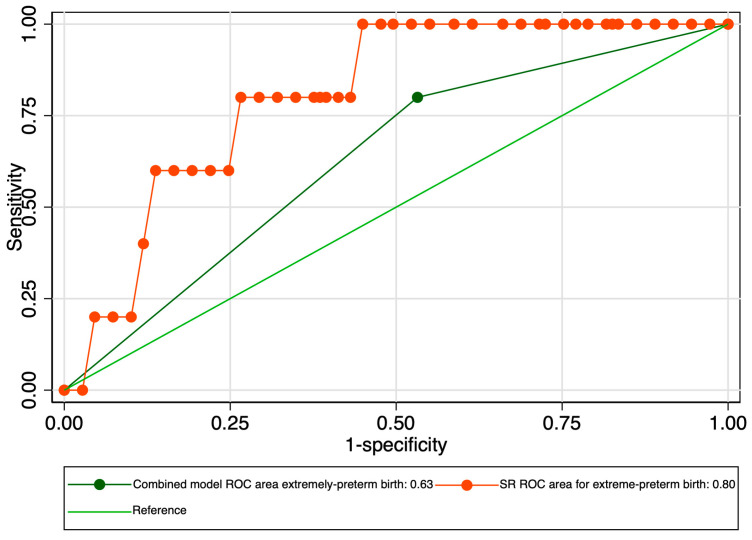
Graphical representation of the comparison between ROC curves for the SR and combined model considering the extremely preterm category.

When evaluating the predictive performance of the elastography marker in the prediction of extremely preterm birth, we obtained an AUROC value of 0.800 (Figure 12). The calculated probability cut-off using the Youden index was 0.63 (Figure 13), and the determined cut-off for this elastography marker using sensitivity analysis was 1.96 (Table 3). The corresponding values for sensitivity, specificity, and precision were 20%, 99%, and 50%, respectively (Table 3). In this context, its negative predictive value (NPV) was high (96.43%), which led to a good overall accuracy (95.61%). The combined model, however, showed a modest performance in terms of the AUROC (0.630), specificity (46.79%), and overall accuracy (48.25%) (Figure 14 and Table 3).

## 4. Discussion

One of the issues facing contemporary obstetricians is identifying pregnant patients who are really at risk of PTB. The predictive performance of current individual markers and combined algorithms is modest, and more in-depth studies on elastography assessment of the uterine cervix, particularly the internal os stiffness, will lead to the eventual adoption of this technique by local hospitals. Cervical elastography has the potential to reduce the need for therapeutic interventions in patients whose risk of PTB is low. This will lessen the needless financial burden involved in managing pregnancies while also improving the comfort of pregnant women.

In this study, we evaluated 114 pregnant patients with a high-risk profile of PTB considering the cervical length and personal comorbidities, who were followed up until birth. Our univariate analysis indicated that women who later delivered prematurely had a shorter cervical length, as well as a personal history of preterm birth, in comparison with controls. All these risk factors were included in the regression analysis of the combined algorithm. Moreover, adverse neonatal outcomes such as rates of NICU admission, need for mechanical ventilation, low birthweight, and low Apgar scores were significantly more prevalent in this group.

The results from our univariate analysis regarding maternal risk factors for PTB and the neonatal outcomes confirm many of the findings published in the current literature. A personal history of PTB is one of the most important risk factors for the recurrence of this event, as demonstrated in a recent analysis of a database comprising 213,335 women [27].

In the second stage of the analysis, we evaluated and compared the predictive performance of the strain ratio at the level of the internal cervical os considering the occurrence of preterm birth and its subtypes. Moreover, we compared its performance to that achieved by a combined algorithm as described above. Our results indicated a good predictive performance of this elastography marker in the prediction of preterm birth before 37 weeks of gestation, and its addition to the combined algorithm resulted in an increase in the overall performance.

When evaluating the predictive performance of this marker in relation to PTB subtypes, we obtained mixed results. Specifically, this marker achieved a low sensitivity and high specificity when taken individually, which indicates that it can correctly identify most patients who will not deliver prematurely. This aspect is extremely important because it can help reduce unnecessary interventions.

On the other hand, the highest AUROC value and accuracy of this marker were achieved in the prediction of extremely preterm birth, which is another important aspect to be taken into consideration because this group of neonates is susceptible to numerous serious complications associated with prematurity and need the most expensive and time-consuming therapeutic interventions. When used in combination with the cervical length and maternal risk factors, the overall accuracy was improved only in the prediction of preterm birth before 37 weeks of gestation, and early preterm birth.

In a prospective nested case–control study by Du et al. that assessed cervical elastography parameters and cervical length during the three trimesters of pregnancy, the authors demonstrated that the strain ratio of the internal os measured in the second trimester of pregnancy was the best predictor of spontaneous PTB, with an AUC value of 0.73, while the cervical length measured in any trimester did not achieve a statistical association with this event [28]. These aspects were confirmed in other observational studies in low- and high-risk populations of pregnant patients [6,29,30].

Similar results were obtained using shear wave elastography in singletons and twin pregnancies. Sun et al. conducted a prospective study that evaluated the predictive performance of this type of elastography in the prediction of PTB in dichorionic diamniotic twin pregnancies [31]. For the mean shear wave elastography of the anterior cervical lip, the authors reported a sensitivity of 83.3%, with a specificity of 57.9%, which confirmed its potential use for the prediction of the evaluated outcome. Another study by Yang et al. assessed the predictive performance of shear wave elastography and cervical length in the prediction of spontaneous PTB in singleton pregnancies, obtaining an AUROC value of 0.98 [32].

A recent systematic review and meta-analysis evaluated the diagnostic accuracy of cervical elastography (both strain and shear wave elastography) in the prediction of PTB [33]. The results indicated an overall AUC value of 0.90 for cervical elastography and 0.60 for cervical length in the prediction of PTB. Therefore, this meta-analysis confirmed the superior predictive performance of cervical elastography and its possible implementation in routine clinical practice.

The majority of previous studies were performed using the semiautomatic tool E-Cervix, while very few studies used the General Electric elastography module. One of these tools was used in a cross-sectional study of 116 pregnant women at 18 to 40 weeks of gestation that assessed the factors affecting the cervical tissue strain [34]. The authors outlined a positive correlation between the cervical strain values at the level of the internal os and gestational age, as well as a negative correlation of this parameter with the cervical length. Another multicentric observational study by Jiang et al. [35], which evaluated the performance and capability of various elastography parameters determined in the second trimester of pregnancy, demonstrated that the strain in the anterior lip of the internal os was an independent predictor of spontaneous preterm birth.

An important problem with elastography is represented by the lack of standardization of the procedure due to the multiple types of analytic software, the lack of associated normograms validated in specific populations, various techniques of transvaginal probe manipulation, strategies for the selection of regions of interest, etc. [36]. For this cohort of patients, we adjusted the strain ratio values to the gestational age at enrollment and calculated specific cut-offs using statistical modeling.

The results of this study must be evaluated considering the following limitations: a small sample size, a lack of specific cut-offs and normograms for the studied population, and a lack of correlation with histological data. On the other hand, its prospective design, the evaluation of the predictive performance of this elastography marker in relation to preterm birth and its subtypes, and the integration of this marker into a combined algorithm constitute strong points of this research. Furthermore, these data can contribute to the limited knowledge in the current literature regarding the applications of the General Electric elastography module for the prediction of preterm birth.

Our results support the idea of external validation of a combined model that includes the values of the strain ratio, cervical length, and maternal characteristics in larger cohorts of patients. Such an approach can help to better quantify the predictive performance of a combined model in heterogenous populations. Moreover, this model could be included in pilot studies for further evaluation, and if its predictive performance is confirmed, it could be implemented in clinical practice, thus reducing costs and unnecessary interventions.

## 5. Conclusions

The strain ratio at the internal cervical os is a reliable tool for the prediction of spontaneous preterm birth before 37 completed weeks of gestation and has a good ability to discriminate most patients who will not deliver prematurely, thus limiting unnecessary therapeutic interventions.

This elastography marker achieved a good predictive performance in the prediction of extremely preterm birth, before 28 weeks of gestation, thus indicating its potential for risk stratification in selected cohorts of patients.

Further studies, on larger cohorts of patients, will be needed to confirm the predictive accuracy of the strain ratio at the internal cervical os in the prediction of preterm birth at various gestational ages.

## Figures and Tables

**Table 1:** Clinical and demographic characteristics of the patients included in the evaluated groups.

Patient’s Characteristics	Group 1 (PTB, *n* = 63)	Group 2 (Controls, *n* = 51)	*p* Value
Age, years (mean ± SD)	30.23 ± 4.16	30.43 ± 4.63	0.48
Medium (*n*/%)	Urban = 29 (46%) Rural = 34 (54%)	Urban = 25 (49%) Rural = 26 (51%)	0.75
BMI, kg/m^2^ (mean ± SD)	22.77 ± 4.98	22.06 ± 3.76	0.28
Smoking (*n*/%)	Yes = 5 (8.2%)	Yes = 6 (12.8%)	0.43
Parity	Nulliparity = 12 (19%) Primiparity = 30 (47.6%) Multiparity = 21 (33.3%)	Nulliparity = 0 (0%) Primiparity = 42 (82.4%) Multiparity = 9 (17.6%)	<0.001
Personal history of preterm birth (*n*/%)	Yes = 12 (19%)	Yes = 0 (0%)	<0.001
Confirmed vaginal infection (*n*/%)	Yes = 21 (33.3%)	Yes = 19 (37.3%)	0.66
Confirmed urinary tract infection (*n*/%)	Yes = 11 (17.5%)	Yes = 10 (19.6%)	0.76
Hypertension (*n*/%)	Yes = 9 (14.3%)	Yes = 3 (5.9%)	0.14
Diabetes (*n*/%)	Yes = 6 (9.5%)	Yes = 3 (5.9%)	0.47
Personal history of adverse pregnancy outcomes (*n*/%)	Yes = 12 (19%)	Yes = 0 (0%)	<0.001
Cervical length, cm (mean ± SD)	1.97 ± 0.67	2.01 ± 0.88	0.03
Cervical funneling (*n*/%)	Yes = 8 (12.69%)	Yes = 0 (0%)	0.03

Table legend: PTB—preterm birth; SD—standard deviation; BMI—body mass index.

**Table 2:** Pregnancy and neonatal outcomes in preterm deliveries.

Outcome	Group 1 (PTB, *n* = 63)	Group 2 (Controls, *n* = 51)	*p* Value
Cesarean delivery (*n*/%)	Yes = 9 (14.28%)	Yes = 1 (1.96%)	0.68
Apgar score at 1 min (mean ± SD)	7.07 ± 2.34	8.70 ± 1.22	0.003
Birthweight, g (mean ± SD)	2516.13 ± 407.28	2911.20 ± 683.04	<0.001
Gender (*n*/%)	Male = 31 (49.2%) Female = 32 (50.8%)	Male = 24 (47.1%) Female = 27 (52.9%)	0.82
NICU admission (*n*/%)	Yes = 9 (14.3%)	Yes = 1 (1.96%)	0.02
Invasive ventilation (*n*/%)	Yes = 8 (12.6%)	Yes = 0 (0%)	0.03
Neonatal death (*n*/%)	Yes = 2 (3.2%)	Yes = 0 (0%)	0.19

Table legend: PTB—preterm birth; SD—standard deviation; NICU—neonatal intensive care unit.

**Table 3:** Predictive performance of simple and combined approaches for the prediction of preterm birth.

Types of PTB	Approach Used	Sensitivity	Specificity	Precision	NPV	FPR	FDR	FNR	Accuracy
PTB before 37 weeks of gestation	SR (cut-off: 0.93)	85.71	84.31	87.10	82.69	15.6	12.9	14.2	85.09
	Combined model	92.31	95.16	94.12	93.65	4	5	7	93.86
PTB between 34 and 37 weeks of gestation	SR (cut-off: 2.56)	5.2	98.9	50	83.9	1	50	94.7	83.3
	Combined model	96.15	24.42	52.63	89.4	72.58	47.37	3.8	58.1
PTB between 28 and 34 weeks of gestation	SR (cut-off: 1.66)	33.33	81.33	48.15	70.11	18.67	51.85	66.6	64.91
	Combined model	98.08	61.29	68	97.44	38.71	32	1	78.07
PTB at less than 28 weeks of gestation	SR (cut-off: 1.96)	20	99	50	96.43	0.9	50	80	95.61
	Combined model	98	6	46.79	80	93.55	53.21	1	48.25

Table legend: PTB—preterm birth; SR—strain ratio at the level of the internal os; NPV—negative predictive value; FPR—false positive rate; FDR—false detection rate; FNR—false negative rate.

## Data Availability

The data presented in this study are available on request from the corresponding author. The data are not publicly available due to local policies.

## References

[1] Oturina V., Hammer K., Möllers M., Braun J., Falkenberg M.K., De Murcia K.O., Schmitz R. (2017). Assessment of cervical elastography strain pattern and its association with preterm birth. J. Perinat. Med..

[2] Mazza E., Parra-Saavedra M., Bajka M., Gratacos E., Nicolaides K., Deprest J. (2014). In vivo assessment of the biomechanical properties of the uterine cervix in pregnancy. Prenat. Diagn..

[3] Jung Y.J., Kwon H., Shin J., Park Y., Heo S.J., Park H.S., Oh S.-Y., Sung J.-H., Seol H.-J., Kim H.M. (2021). The Feasibility of Cervical Elastography in Predicting Preterm Delivery in Singleton Pregnancy with Short Cervix Following Progesterone Treatment. Int. J. Environ. Res. Public. Health.

[4] Gesthuysen A., Hammer K., Möllers M., Braun J., Oelmeier de Murcia K., Falkenberg M.K., Köster H.A., Möllmann U., Fruscalzo A., Bormann E. (2020). Evaluation of Cervical Elastography Strain Pattern to Predict Preterm Birth. Ultraschall Med..

[5] Nazzaro G., Saccone G., Miranda M., Crocetto F., Zullo F., Locci M. (2022). Cervical elastography using E-cervix for prediction of preterm birth in singleton pregnancies with threatened preterm labor. J. Matern. Fetal Neonatal Med..

[6] Woźniak S., Czuczwar P., Szkodziak P., Wrona W., Paszkowski T. (2015). Elastography for predicting preterm delivery in patients with short cervical length at 18–22 weeks of gestation: A prospective observational study. Ginekol. Pol..

[7] Hernandez-Andrade E., Garcia M., Ahn H., Korzeniewski S.J., Saker H., Yeo L., Chaiworapongsa T., Hassan S.S., Romero R. (2015). Strain at the internal cervical os assessed with quasi-static elastography is associated with the risk of spontaneous preterm delivery at ≤34 weeks of gestation. J. Perinat. Med..

[8] Beta J., Akolekar R., Ventura W., Syngelaki A., Nicolaides K.H. (2011). Prediction of spontaneous preterm delivery from maternal factors, obstetric history and placental perfusion and function at 11–13 weeks. Prenat. Diagn..

[9] Celik E., To M., Gajewska K., Smith G.C., Nicolaides K.H. (2008). Cervical length and obstetric history predict spontaneous preterm birth: Development and validation of a model to provide individualized risk assessment. Ultrasound Obs. Gynecol..

[10] Damaso E.L., Rolnik D.L., Cavalli R.C., Quintana S.M., Duarte G., da Silva Costa F., Marcolin A. (2019). Prediction of Preterm, B.irth by Maternal Characteristics and Medical History in the Brazilian Population. J. Pregnancy.

[11] Vicoveanu P., Vasilache I.A., Nemescu D., Carauleanu A., Scripcariu I.S., Rudisteanu D., Burlui A., Rezus E., Socolov D. (2022). Predictors Associated with Adverse Pregnancy Outcomes in a Cohort of Women with Systematic Lupus Erythematosus from Romania—An Observational Study (Stage 2). J. Clin. Med..

[12] Vicoveanu P., Vasilache I.A., Scripcariu I.S., Nemescu D., Carauleanu A., Vicoveanu D., Covali A.R., Filip C., Socolov D. (2022). Use of a Feed-Forward Back Propagation Network for the Prediction of Small for Gestational Age Newborns in a Cohort of Pregnant Patients with Thrombophilia. Diagnostics.

[13] Radu V.D., Vasilache I.A., Costache R.C., Scripcariu I.S., Nemescu D., Carauleanu A., Nechifor V., Groza V., Onofrei P., Boiculese L. (2022). Pregnancy Outcomes in a Cohort of Patients Who Underwent Double-J Ureteric Stenting—A Single Center Experience. Medicina.

[14] Săndulescu M.S., Văduva C.C., Siminel M.A., Dijmărescu A.L., Vrabie S.C., Camen I.V., Tache D.E., Neamţu S.D., Nagy R.D., Carp-Velişcu A. (2022). Impact of COVID-19 on fertility and assisted reproductive technology (ART): A systematic review. Rom. J. Morphol. Embryol..

[15] Van Zijl M.D., Koullali B., Mol B.W.J., Snijders R.J., Kazemier B.M., Pajkrt E. (2020). The predictive capacity of uterine artery Doppler for preterm birth-A cohort study. Acta Obs. Gynecol. Scand..

[16] Camen I.V., Manolea M.M., Vrabie S.C., Sandulescu M.S., Serbanescu M.S., Boldeanu M.V., Novac M.B. (2022). The Ability of Doppler Uterine Artery Ultrasound to Predict Premature Birth. Curr. Health Sci. J..

[17] Bernad S.E., Barbat T., Barbu D., Albulescu V. (2010). Assessment of the placental blood flow in the normally developing and growth-restricted fetus. Advances in Perinatal Medicine.

[18] Grobman W.A., Lai Y., Iams J.D., Reddy U.M., Mercer B.M., Saade G., Tita A.T., Rouse D.J., Sorokin Y., Wapner R.J. (2016). Prediction of Spontaneous Preterm Birth among Nulliparous Women with a Short Cervix. J. Ultrasound Med..

[19] Guerby P., Girard M., Marcoux G., Beaudoin A., Pasquier J.C., Bujold E. (2023). Midtrimester Cervical Length in Low-Risk Nulliparous Women for the Prediction of Spontaneous Preterm Birth: Should We Consider a New Definition of Short Cervix?. Am. J. Perinatol..

[20] Singh P.K., Srivastava R., Kumar I., Rai S., Pandey S., Shukla R.C., Verma A. (2022). Evaluation of Uterocervical Angle and Cervical Length as Predictors of Spontaneous Preterm Birth. Indian J. Radiol. Imaging.

[21] O’Hara S., Zelesco M., Sun Z. (2013). Cervical length for predicting preterm birth and a comparison of ultrasonic measurement techniques. Australas. J. Ultrasound Med..

[22] Suresh S., MacGregor C., Dude A., Hirsch E. (2022). Single second-trimester cervical length is predictive of preterm delivery among patients with prophylactic cerclage. Am. J. Obstet. Gynecol..

[23] Lucaroni F., Morciano L., Rizzo G., Antonio F.D., Buonuomo E., Palombi L., Arduini D. (2018). Biomarkers for predicting spontaneous preterm birth: An umbrella systematic review. J. Matern. Fetal Neonatal Med..

[24] Hornaday K.K., Wood E.M., Slater D.M. (2022). Is there a maternal blood biomarker that can predict spontaneous preterm birth prior to labour onset? A systematic review. PLoS ONE.

[25] Vogel J.P., Chawanpaiboon S., Moller A.B., Watananirun K., Bonet M., Lumbiganon P. (2018). The global epidemiology of preterm birth. Best Pract. Res. Clin. Obs. Gynaecol..

[26] Karnati S., Kollikonda S., Abu-Shaweesh J. (2020). Late preterm infants–Changing trends and continuing challenges. Int. J. Pediatr. Adolesc. Med..

[27] Tingleff T., Vikanes Å., Räisänen S., Sandvik L., Murzakanova G., Laine K. (2022). Risk of preterm birth in relation to history of preterm birth: A population-based registry study of 213,335 women in Norway. BJOG Int. J. Obstet. Gynaecol..

[28] Du L., Zhang L.H., Zheng Q., Xie H.N., Gu Y.J., Lin M.F., Wu L. (2020). Evaluation of Cervical Elastography for Prediction of Spontaneous Preterm Birth in Low-Risk Women: A Prospective Study. J. Ultrasound Med..

[29] Światkowska-Freund M., Traczyk-łoś A., Preis K., łukaszuk M., Zielińska K. (2014). Prognostic value of elastography in predicting premature delivery. Ginekol. Pol..

[30] Wozniak S., Czuczwar P., Szkodziak P., Milart P., Wozniakowska E., Paszkowski T. (2014). Elastography in predicting preterm delivery in asymptomatic, low-risk women: A prospective observational study. BMC Pregnancy Childbirth.

[31] Sun J., Li N., Jian W., Cao D., Yang J., Chen M. (2022). Clinical application of cervical shear wave elastography in predicting the risk of preterm delivery in DCDA twin pregnancy. BMC Pregnancy Childbirth.

[32] Yang X., Ding Y., Mei J., Xiong W., Wang J., Huang Z., Li R. (2022). Second-Trimester Cervical Shear Wave Elastography Combined with Cervical Length for the Prediction of Spontaneous Preterm Birth. Ultrasound Med. Biol..

[33] Wang B., Zhang Y., Chen S., Xiang X., Wen J., Yi M., Hu B. (2019). Diagnostic accuracy of cervical elastography in predicting preterm delivery A systematic review and meta-analysis. Medicine.

[34] Wongsaroj P., Moungmaithong S. (2017). Cervical Strain Values Measured by Ultrasonographic Elastography in Pregnant Women between 18 and 40 Weeks’ Gestation. J. Med. Assoc. Thai.

[35] Jiang L., Peng L., Rong M., Liu X., Pang Q., Li H., Wang Y., Liu Z. (2022). Nomogram Incorporating Multimodal Transvaginal Ultrasound Assessment at 20 to 24 Weeks’ Gestation for Predicting Spontaneous Preterm Delivery in Low-Risk Women. Int. J. Womens Health.

[36] Swiatkowska-Freund M., Preis K. (2017). Cervical elastography during pregnancy: Clinical perspectives. Int. J. Womens Health.

